# Counting Highly Cited Papers for University Research Assessment: Conceptual and Technical Issues

**DOI:** 10.1371/journal.pone.0047210

**Published:** 2012-10-12

**Authors:** Alonso Rodríguez-Navarro

**Affiliations:** Centro de Biotecnología y Genómica de Plantas, Universidad Politécnica de Madrid, Madrid, Spain; Tel Aviv University, Israel

## Abstract

A Kuhnian approach to research assessment requires us to consider that the important scientific breakthroughs that drive scientific progress are infrequent and that the progress of science does not depend on normal research. Consequently, indicators of research performance based on the total number of papers do not accurately measure scientific progress. Similarly, those universities with the best reputations in terms of scientific progress differ widely from other universities in terms of the scale of investments made in research and in the higher concentrations of outstanding scientists present, but less so in terms of the total number of papers or citations. This study argues that indicators for the 1% high-citation tail of the citation distribution reveal the contribution of universities to the progress of science and provide quantifiable justification for the large investments in research made by elite research universities. In this tail, which follows a power low, the number of the less frequent and highly cited important breakthroughs can be predicted from the frequencies of papers in the upper part of the tail. This study quantifies the false impression of excellence produced by multinational papers, and by other types of papers that do not contribute to the progress of science. Many of these papers are concentrated in and dominate lists of highly cited papers, especially in lower-ranked universities. The *h*-index obscures the differences between higher- and lower-ranked universities because the proportion of *h-*core papers in the 1% high-citation tail is not proportional to the value of the *h*-index.

## Introduction

“Government policy-makers, corporate research managers, and university administrators need valid and reliable S&T indicators for a variety of purposes: for example, to measure the effectiveness of research expenditures, identify areas of strength and excellence, set priorities for strategic planning, monitor performance relative to peers and competitors, and target emerging specialties and new technologies for accelerated development.” So begins a paper by Garfield and Welljams-Dorof [1], and the essence of this idea can be found in the introductions to countless papers published before and since. Consistent with this idea, many research indicators have been developed (see, for example [2]–[4]). It is unclear, however, whether the indicators currently used accurately measure all that governments and research administrators need to know, or whether such indicators are always correctly interpreted and applied by governments and research administrators [5]–[7].

The Spanish government, for example, recently announced that the quality of scientific research in Spain had overtaken that of Switzerland [8], but this statement is inconsistent with the role research plays in the economic realities of the respective countries. While the responsibility for any misstatement would lay exclusively with its author, if it is based on a research indicator–in this case, citation counts of all published papers–the validity of that indicator as a numerical measure of research performance should be brought into question. The use of indicators of research performance based on measures such as the numbers of all papers published and their subsequent citations contributes to not only misleading conclusions about a country’s research performances but also to the notion that elite research institutions are not using their research investment funds in an efficient manner.

Considering the highest- and lowest-ranked universities in [9], MIT’s research revenues exceed 1.3 billion US dollars per year (http://web.mit.edu/facts/financial.html, accessed on August 2011), whereas the equivalent figure for Complutense University of Madrid is 21 million euros (approximately, 27 million US dollars; http://www.redotriuniversidades.net/, accessed on August 2011). The comparison of these figures leads to the conclusion that the difference in research performance between these two universities is not best indicated by the ratio of the number of publications or of the other indicators based on the total number of publications, which may have values of 2–3 (for example: *Academic Ranking of World Universities 2010*, PUB score, http://www.arwu.org/ARWU2010.jsp, accessed on August 2011; *Excellence Rate Report*, http://www.scimagoir.com, downloaded November, 2011). It is, of course, difficult to make comparisons regarding research funding and output [10], and the differences in the accounting methods across institutions must be corrected before making comparisons. However, even having compensated for those differences, the investment ratio between MIT and Complutense University is still greater than 10∶1. In addition, the high number of researchers having received Nobel prizes or other awards [11] and with a high number of citations [12] at MIT suggests that the institution tends to hire high-level researchers in contrast with the suboptimal methods for researcher selection to which Spanish universities adhere [13]. All this suggests that differences in research performance between MIT and Complutense University should be even greater than those suggested by the differences in funding and ought not to be expressed as simply a ratio of 3.0. Another way to view this issue is calculating the ratio of research investment per paper for the two institutions. This calculation reveals that the MIT’s cost of one paper is seven times higher than in Complutense University, which suggests that those papers coming out of MIT are not comparable to those coming out of Complutense University.

These examples illustrate that some research indicators may be problematic at both country and university levels. Although the problems can be investigated at both levels, the university level is easier to investigate because universities are research units more homogeneous than countries, where very different institutions coexist. Moreover, it is easy to select a sample of universities that are very different in research activity and similar in size to simplify the analysis of the results.

To evaluate overall research performance, the *x*-index was recently formulated using a statistical procedure which optimized the correlation of the indicator with the number of Nobel Prize achievements [9]. The *x*-index only considers the papers that are included in the world’s top 1% of cited papers [9] and not all these papers. In particular, multinational and review papers are not counted, and a subtraction term is included in the formula to statistically discount the papers that report methods, clinical trials, and statistics (MCTS papers). Although the statistical procedure to formulate the *x*-index was effective, it does not address conceptual questions about the papers that are not counted. Besides, it does not provide any indication about the number of papers that are counted with reference to the total number of papers in universities of different research levels. However, this is information is necessary to establish a solid scientific background for evaluations based on the world’s top 1% of cited papers.

Considering these issues, this study aims to answer three specific questions regarding the *x*-index, or any other research indicator based on highly cited papers: (i) Is the exclusion of 99% of the published papers supported by conceptual or empirical reasons? (ii) Which is the proportion of multinational, reviews, and MCTS papers in highly cited papers? (iii) Is it possible to simplify the *x*-index formula for universities? The first question is at the basis of research evaluations and it will be addressed from a general point of view. The other two questions are purely technical and they will be addressed studying 18 universities of different research levels.

### The Basis of Research Evaluation: A Kuhnian View of Research Performance

The majority of research papers that are published every year receive an ephemeral attention from researchers and no attention from the society, but nevertheless, relevant papers are somehow dependent from apparently irrelevant papers. In principle, this situation resembles a soccer match, where many passes are necessary to strike a goal. The number of passes can be counted, but this number does not determine the winner of the match, which is determined by counting the very low frequency events in which the ball is kicked to the goal. Similarly in research, many papers are necessary to make discoveries, but very few report actual discoveries. Unlike soccer, however, in research, there are discoveries of different levels that cannot be easily counted or added up together. Therefore, valid and reliable indicators are required to estimate the capacity of countries and institutions to drive scientific progress.

Output indicators of scientific research performance were developed many years ago [14]. Although there are many types, most such indicators are based on the total number of published papers. At a first view, this approach seems to be in contrast with the notion that science does not progress linearly, via a steady accumulation of published information. Early views of this notion were established by James McKeen Cattell (for a review see [15]) and by José Ortega y Gasset [16], but it was Thomas Kuhn [17] who formalized it in his seminal work, *The Structure of Scientific Revolutions*, in this study coining the terms “normal” and “revolutionary” sciences. Kuhn demonstrated that only revolutionary science exerts change on the fundamental structures of science through “paradigm shifts.” As an extension of this central idea, it may be added that, in the short run, important paradigm extensions drive a type of scientific progress that the society appreciates and that must be considered to estimate research performance.

An illustrative example of scientific progress that is based on paradigm extension can be taken from a cancer treatment breakthrough, the use of imatinib mesyalate in the treatment of chronic myelogenous leukemia. The excellent clinical performance of this drug is based on two biological features: a chromosomal translocation that creates a fusion gene, which is vital for the expansion of cancer cells, and the inhibition of the product of this gene by imatinib [18]. The discoveries of these features constituted important breakthroughs in cancer research, but neither discovery represented a paradigm shift. Even the concept of a drug able to kill cancer cells without damaging normal cells is only an extension of the “magic bullet” concept popularized by Paul Ehrlich one hundred years ago [19]. The paradigm shift occurred when Paul Ehrlich developed Salvarsan to treat syphilis and not with the present-day development of imatinib.

This example can also be used to answer the question of whether the total number of papers published by countries and institutions should be considered as an accurate means of evaluating their research performance. More than 120,000 papers are published annually on cancer research [20], but drugs with the properties of imatinib constitute exceptional discoveries. The contributions made by countries and institutions to the total number of papers published on cancer research are therefore unlikely to be a sound indicator of their actual contribution to the progress of cancer treatment. For this to be correct, breakthroughs should occur in proportion to the total number of papers published across all countries and institutions, a condition which has never been demonstrated.

The discussion of indicators of research performance is more complex than just a numerical comparison as many of the total number of papers are important and necessary to researchers even if these papers do not directly report on scientific progress. In fact, researchers value these papers. However, the larger society, which pays for the research, is interested in tangible evidence of progress, in both technological and basic research, not in the intermediate steps (this is a short description of a complex problem; see, for example [5], [21], [22]). Many of these intermediate steps may be considered exploratory research that leads nowhere. Research in the natural sciences is full of exciting lines of investigation that are, in the end, abandoned and superseded by others that are often completely different. Going back to the example of syphilis, the first treatment applied was mercury, which was replaced by bismuth, which was replaced by Salvarsan, which was replaced by penicillin; bismuth is being used again, now to kill the bacterium that causes gastric ulcers [23]. Research on the chemotherapeutic properties of mercury would be unjustified in light of current knowledge, but a similar conclusion does not apply to bismuth. These details of the discovery process are complex and rarely of interest to the wider society, which needs solutions rather than results of exploratory research of possible solutions with unknown probabilities of success.

According to this line of reasoning, indicators of research performance should reflect the substantive contributions made to scientific progress; the relative rates at which two institutions make important discoveries should be considered first in comparing their scientific performance. Therefore, papers reporting real scientific progress should be the basis of research evaluations, even though these papers represent but a small proportion of all published papers. The higher the proportion of these papers, the better rated the research performance. It must be underscored, however, that this conclusion applies to institutions with many researchers. In evaluating individual researchers, the analysis must be different, and the contributions made to the intermediate steps of research must be considered. The main reason is statistical, because important discoveries are low frequency events and even the most highly capable researchers are not assured of achieving even a single such discovery. The capability of a researcher is therefore established as a probability of achieving an important discovery, which can in turn be estimated from the researcher’s success in normal research.

The only practical approach to estimating the number of papers that report scientific progress is citation counts, despite the number of technical questions the method raises [24]. Assuming that papers which report important breakthroughs are highly cited–as is demonstrated, for instance, by the high number of citations of the crucial papers of Nobel Prize winners [25], [26]–the resulting working hypothesis is that indicators of scientific performance for countries and institutions should be based on highly cited papers. If this hypothesis were correct, indicators that consider the total number of papers might give an erroneous estimation of research performance. Exceptionally, this estimation would be correct in countries with similarly efficient research systems [27].

## Methods

**Figure 1:**
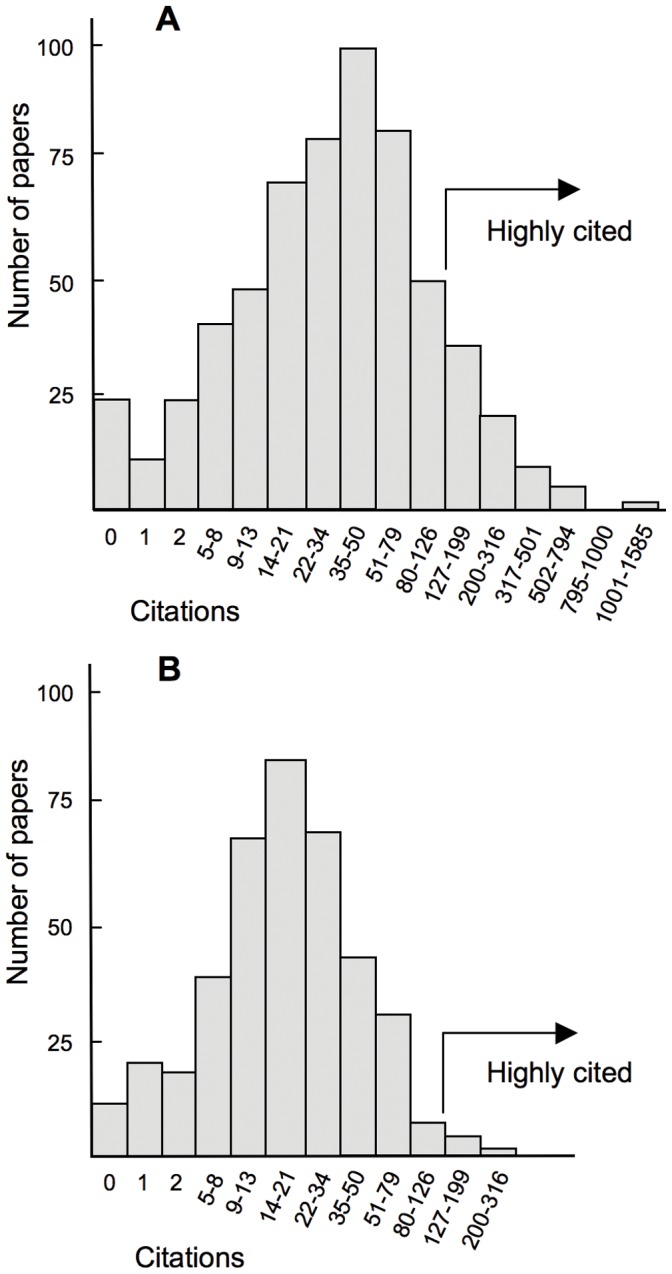
Frequency distribution of citations to the scientific publications in chemistry of two universities. MIT (**A**) and Complutense University of Madrid (**B**) in 2002 and 2003 in the field of chemistry. The number of citations of the papers published in two years are recorded together and plotted using a logarithmic scale for citations. Publications with more than 126 citations are marked as highly cited.

The data-collection methods used in this study were described in a previous paper [9]. In brief, I used the Web of Science database, restricted to the Science Citation Index Expanded database, and the Essential Science Indicators^SM^ (ESI) resource in Thomson Reuters’ ISI Web of Knowledge (http://isiknowledge.com). To retrieve national publications (single-country papers with authors from the involved university), the name of the university was entered in the “Address” search field with the name of the corresponding country using the Boolean operator “SAME” and followed by the names of the remaining 22 countries with the highest number of publications according to the ESI using the Boolean operator “NOT”. To restrict the search to research articles, the option “Article” was selected in the “Document Type” search field. Most searches were restricted to a single year using the “Year Published” search field. The minimum number of citations needed for the publications of a certain year to belong to the percentile ranges 1% and 0.1% are recorded in the percentiles table of the Baselines menu of the ESI. For this study, except in Figure 1 and Table 1, the percentile breakdowns for “All Fields” were used. After each search, the retrieved papers were sorted by the number of times cited, starting with the most-cited paper, and the number of papers in each percentile was determined by the rank number of the last paper that had the required minimum number of citations according to the percentiles table in the ESI Baselines menu. When necessary, a Marked List was created for these papers. Next, the total number of citations and average citations per item were obtained using the “Create Citation Report” feature.

**Table 1:** Scientific publications of MIT and Complutense University of Madrid in 2002 and 2003 in the field of chemistry: number of papers in top citation percentiles.

University	All papers	10%	1%	0.1%
MIT	616	202	36	7
CUM	422	18	1	0

Papers in top citation percentiles were identified independently for 2002 and 2003; the resulting figures were then summed.

The data summarized in Figure 1 and Table 1 were obtained using the Subject Area feature of the Advanced Search of the Web of Science, SU = (Chemistry). The data were drawn from two consecutive years, 2002 and 2003, to increase the number of papers included in the sample. In Figure 1 the data of the two years are shown together; in Table 1 the data of the two years were treated independently and the results were then added together. Approximately half of the papers recorded by the Science Citation Index Expanded database for MIT in chemistry are Abstracts of Papers of the American Chemical Society with zero citations; these papers were not counted.

Except for Figure 1 and Table 1, all of the bibliometric data reported in this study were obtained between August 8 and August 20, 2011. During these searches the ESI database was updated as of July 1, 2011. The data recorded in Figure 1 and Table 1 were obtained in June 2012.

## Results

### Frequency of Important Breakthroughs

The conclusion that indicators of research performance for countries and institutions should be based on highly cited papers raises a question about the citation level of these papers. This level is important because it determines the proportion of papers that are included in the evaluation, which involves the first question of this report.

As a case study meant to facilitate an examination of this proportion and the associated number of citations of the involved papers, I will use the abovementioned example of the treatment of chronic myelogenous leukemia. I chose to analyze papers published in 2001, as this was the year in which the most-cited paper about the research, which led to the discovery of imatinib [29], was published. The selected case study presented a technical problem in that papers on this topic are distributed across two fields of research in the ESI database, Clinical Medicine and Molecular Biology & Genetics, which have different percentile breakdowns. In the less restrictive field, Clinical Medicine, the 1% and 0.1% breakdowns for 2001 were 192 and 585 citations, respectively, and I used these breakdowns. A search for the topic “leukemia” retrieved 8,247 papers, of which 631 were reviews and 5,829 were articles. The 8,247 papers received 218,945 citations (mean = 26.6). The most-cited paper was that already mentioned [29], which was cited 2,150 times. There were 17 papers in the top 0.1% of highly cited papers, which received a total of 18,761 citations (mean = 1,104), and 120 papers in the top 1% of highly cited papers, which received a total of 48,274 citations (mean = 402). The interesting scientometric question is how many of these published papers report on important breakthroughs, whether they represent scientific or clinical advances. I did not try to answer this question by conducting a survey among researchers. However, considering the activity in the field and the major repercussions of any significant advance in publications about cancer research, I would expect that no papers that did not reach the top 0.1%, and perhaps not even all the papers in that percentile, reported important breakthroughs. According to this estimate, only 0.2% of the published papers on leukemia in 2001 may have reported important breakthroughs; although these papers were very highly cited, they received only 8.6% of the citations of all papers.

These small percentages demonstrate that indicators based on the total number of papers may fail completely to recognize the institutions that make greater contributions to the field of leukemia research.

### Comparing Two Very Different Universities

A more general approach to address the question about the citation level that delimits the papers that should be counted for research evaluations is the comparison of two universities with very different levels of research performance. For this purpose, the two universities that I compared above, MIT and Complutense University of Madrid, meet the indicated requirements. Considering that the ratio between research investments is in excess of 10∶1 and taking into account the expected performance of researchers, a reliable indicator of research performance could be as great as 50 times higher for MIT than for Complutense University.

To perform the study I chose the field of chemistry because projects of “big science” and papers involving authors from many countries are less frequent in this field than they are in physics or biology. Comparing MIT to Complutense University, the ratios between the total number of papers, 616 versus 422; or citations, 37,701 versus 8,154; or the mean number of citations per paper, 61.1 versus 19.3, varied from 1.5 to 4.6, all figures that are far from the abovementioned value of 50. This finding indicates that indicators calculated from all published papers are unlikely to reveal the actual research performance of universities. A comparison of the frequency distribution of citations (Figure 1 and Table 1) indicates that even considering the share of world’s top 1% of cited papers, the expected differences between MIT and Complutense University are not clearly revealed.

### Basic Data of 18 Universities

Next, I selected 18 universities of different countries and research levels according to SCImago and CWTS university rankings, including three US universities: MIT, Cornell University, and The University of Utah. All universities included in the study are well-known universities in their respective countries but are not necessarily the highest ranked institutions in each country. The Indian Institute of Technology consists of several autonomous universities; data for the institute as a whole were used for this study.

Table 2 summarizes the research activities of the selected universities in terms of the number of papers published in 2005 and the number of citations of those papers as of the search date; Table 3 summarizes the corresponding parameters for the papers of each university in the world’s top 1% of cited papers in 2005. Both tables show national and multinational papers separately. Ordering the universities using the number of citations, the resultant ranking lists were highly similar; both tables are shown ordered as in Table 3 concerning national papers. The most striking difference between the two tables was the variation in the number of citations from the most- to the least-cited university. For example, in Table 2, national papers, the number of citations varied from 103,095 for MIT to 10,790 for the National Autonomous University of México; the corresponding highest and lowest values in Table 3 were from 46,165, for MIT, to 317, for the National Autonomous University of México. In terms of the number of papers, the variation was again larger in Table 3. For example, the number of national papers in Table 2 varied from a high of 7,219, for Osaka, to a low of 1,164 for Paris-Sud 11; the corresponding highest and lowest values in Table 3 were 158 for MIT and 2 for the National Autonomous University of México, respectively.

**Table 2:** Scientific publications from 18 universities in 2005: number of papers and citations, and mean number of citations per paper.

	National publications			Multinational publications			Ratio (Multinat./Nat.)	
University	Papers	Citations	Mean	Papers	Citations	Mean	Papers	Citations
MIT	3,980	103,095	25.9	1,310	49,828	38.0	0.33	0.35
Cornell	4,946	77,057	15.6	1,164	36,674	31.5	0.24	0.48
Oxford	4,779	70,745	14.8	2,496	76,810	30.8	0.52	1.09
Kyoto	6,271	77,576	12.4	1,353	31577	23.3	0.21	0.41
Toronto	6,649	75,435	11.4	3,135	76,759	24.5	0.47	1.02
Osaka	7,219	73,369	10.2	1,415	33,022	23.3	0.20	0.45
Utah	3,300	43,338	13.1	678	18,369	27.1	0.21	0.42
Stockholm	1,942	29,774	15.3	1,147	29,804	26.0	0.59	1.00
Heidelberg	2,525	31,796	12.6	1,233	37,462	30.4	0.49	1.18
Seoul Nat.	3,959	37,829	9.6	1,051	21,288	20.3	0.27	0.56
Utrecht	2,732	36,424	13.3	1,357	35,071	25.8	0.50	0.96
ETH Zurich	1,516	23,099	15.2	1,303	31,477	24.2	0.86	1.36
Melbourne	3,975	38,442	9.7	1,476	32,261	22.1	0.37	0.85
Sapienza	2,743	26,355	9.6	1,032	22,490	21.8	0.45	0.97
Indian Inst. Technology	3,264	25,290	7.8	591	8,965	15.2	0.18	0.35
Paris-Sud 11	1,164	13,652	11.7	914	23,369	25.6	0.79	1.71
Complutense^a^	1,485	15,415	10.4	410	7,408	18.1	0.31	0.64
Natl. Auton. México^b^	1,739	10,790	6.2	892	11,892	13,3	0.51	1.10

National and multinational papers are separated, and the corresponding ratios for the number of papers and citations are recorded.

a Complutense University of Madrid;

b National Autonomous University of México.

The mean number of citations showed low variability across universities in all cases for both Tables 2 and 3, national and multinational papers. The mean number of citations for the papers that were not in the top 1% of highly cited papers can be calculated by subtracting the numbers of papers and the numbers of citations recorded in Tables 2 and 3 and dividing the respective differences. These means showed very low variation between the top and bottom universities. For national papers, for example, the means were 14.9 and 12.1 for MIT and Cornell, respectively, versus 10.8 and 10.0 for Paris-Sud 11 and Complutense, respectively.

**Table 3:** Scientific publications among the world’s top 1% of highly cited papers from 18 universities in 2005: number of papers, citations, and mean number of citations per paper.

	National publications			Multinational publications			Ratio (Multinat./Nat.)	
University	Papers	Citations	Mean	Papers	Citations	Mean	Papers	Citations
MIT	158	46,165	292	88	24,570	279	0.56	0.53
Cornell	97	18,241	188	64	15,964	249	0.66	0.88
Oxford	79	17,466	221	115	30,811	268	1.46	1.76
Kyoto	56	12,864	230	34	9,313	274	0.61	0.72
Toronto	59	10,117	172	105	24,632	235	1.78	2.43
Osaka	39	9,721	249	51	12,213	240	1.31	1.26
Utah	50	8,194	164	23	7,550	328	0.46	0.92
Stockholm	25	5,808	232	45	9,651	214	1.80	1.66
Heidelberg	22	4,629	210	47	14,117	300	2.14	3.05
Seoul National	24	4,226	176	24	5,618	234	1.00	1.33
Utrecht	26	4,209	162	43	11,208	261	1.65	2.66
ETH Zurich	18	2,996	166	38	8,324	219	2.11	2.78
Melbourne	18	2,527	140	38	8,209	216	2.11	3.25
Sapienza	13	2,461	189	27	5,725	212	2.08	2.33
Indian Inst. Technology	10	1,773	182	10	2,355	236	1.00	1.33
Paris-Sud 11	8	1,069	134	29	8,573	296	3.63	8.02
Complutense^a^	4	614	154	7	1,732	247	1.75	2.82
Natl. Aunton. México^b^	2	317	159	6	886	148	3.00	0.93

National (single-country) and multinational publications were counted separately; the corresponding ratios for the two categories of papers are recorded in the last two columns.

a Complutense University of Madrid;

b National Autonomous University of México.

### Multinational Papers

Multinational papers are omitted in the formula of the *x*-index [9]. Integer counting of multinational papers is frequently used in bibliometric studies (e.g., [28]), although the method is formally incorrect because it inflates the paper count. For example, if 50 universities from 10 countries collaborated on a published paper, assigning the paper once to each country and institution would count the paper ten times in country rankings and fifty times in institution rankings. For country evaluations, fractional counting–i.e., allotting fractions of multinational papers and of the total count of citations to each participating institutions–corrects the inflation effect of integer counting on bibliometric indicators. However, there is no way to correct for differences in scientific leadership (see Discussion, below).

The obscuring effect of integer counting on research indicators can be estimated by comparing the weight of multinational papers in the bibliometric parameters. The effect was minor when all the papers were considered, given that the ratio between the numbers of multinational and national papers was low. For the 18 universities included in this study the multinational to national ratio for all papers varied from 0.21 for Kyoto and Utah up to 0.86 for ETH, i.e., most or the papers are national. In contrast, for papers in the top 1% of highly cited papers, the corresponding ratio varied from 0.46 for Utah to 3.63 for Paris-Sud 11, i.e., most of the papers were multinational in Paris-Sud 11. The problem with regard to the number of citations is similar, but the differences are even more striking. For papers in the top 1% of highly cited papers, the ratio between citations to multinational and national papers varied form 0.53 for MIT, to 8.02 for Paris-Sud 11. The multinational to national ratio in the top 1% of highly cited papers was dependent on the relative rank of the universities. In lower-ranked universities the weight of multinational papers in each of the two indicators, number of papers and number of citations, was greater than for the higher-ranked universities (compare corresponding ratios in Table 2 and 3).

A final observation regarding the top 1% of highly cited papers was that the number of institutions participating in multinational papers was considerably higher than that in national papers. For example, for Sapienza, Paris-Sud 11, and Complutense, the mean numbers of institutions participating in national papers were, 4.9, 3.8, and 1.4, respectively, while the means for multinational papers were 17, 13 and 10, respectively.

### Review Papers

Review papers were also concentrated in the top 1% of highly cited papers. Table 4 provides the numbers of national papers in this percentile and the number of citations to these papers, as retrieved from the Web of Science database using the “Document Types” options of “Article” or “Review” (adding the two figures given yields the number of national papers provided in Table 3). The data in Table 4 reveal that review papers comprised a significant proportion of the papers in the top 1% of highly cited papers; this proportion, however, was uneven across universities. Review papers made up only 17% of the national papers in MIT, but that figure was 39% for ETH, 37% for Toronto University, 36% for Stockholm University, and 31% for Oxford University. In general, the mean number of citations of review papers was higher than that of articles, but it was neither much higher nor always higher.

**Table 4:** National scientific publications among the world’s top 1% of highly cited papers from 18 universities in 2005 recorded in the database as articles or reviews: number of papers, citations, and mean number of citations per paper.

	Articles			Reviews		
University	Papers	Citations	Mean	Papers	Citations	Mean
MIT	131	40,307	308	27	5,858	217
Cornell	71	13,244	187	26	4,997	192
Oxford	54	11,738	217	25	5,728	229
Kyoto	48	10,082	210	8	2,782	348
Toronto	37	6,390	173	22	3,727	169
Osaka	32	7,457	233	7	2,264	323
Utah	40	6,420	161	10	1,774	177
Stockholm	16	2,711	169	9	3,097	344
Heidelberg	14	2,911	208	8	1,718	215
Seoul National	20	3,227	161	4	999	250
Utrecht	21	3,464	165	5	745	149
ETH Zurich	11	1,840	167	7	1,156	165
Melbourne	14	1,938	138	4	589	147
Sapienza	10	1,781	178	3	680	227
Indian Inst.Technology	5	684	137	5	1,089	218
Paris-Sud 11	6	848	141	2	221	111
Complutense^a^	3	477	159	1	137	137
Natl. Auton.México^b^	0	0	–	2	317	159

a Complutense University of Madrid;

b National Autonomous University of México.

### Methods, Clinical-trials, and Statistical Papers

To correct the problem created by MCTS papers, the formula of the *x*-index contains a subtraction term, but this term gave rise to negative index values for countries with less-competitive research systems [9]. Returning to the example used above in the field of cancer research, the problem introduced by these papers can be demonstrated with the discovery of imatinib, likely the most important advance in cancer research in many years. Notably, the most highly cited paper regarding this drug [29] received 2,150 citations since its date of publication in 2001. This number is only 27 more citations than have been received by a cancer statistics paper from the same year [30], and 2,275 fewer citations than have been received by a cancer statistics paper from 2008 [31].

As described for multinational and review papers, MCTS papers are concentrated in the top 1% of highly cited papers. However, unlike multinational and review papers, it is difficult if not impossible to identify and count MCTS papers. Among similarly sized institutions MCTS papers fortunately operate as a constant addition term in the *x*-index formula. If this term is omitted the correlation coefficient between the *x*-index and the number of Nobel Prizes decreases a little and the ranking order is not affected. I examined the concentration of MCTS papers among those papers published by universities and concluded that the effects are a minor problem for high- or mid-ranking US universities but are more important to European universities. Although identifying MCTS papers by reading abstracts was not easy, I believe that they account for a higher percentage of the highly cited papers for countries than they do for universities, perhaps due to the large number of hospitals and government agencies which produce clinical trials and statistical studies.

In summary, to rank universities the subtraction term in the formula of the *x*-index can be eliminated. Although this new *x*-index for universities will overvalue some lower-ranked universities, the problem created by MCTS papers does not have a better solution.

### The Top 1% and 0.1% of Highly Cited Papers

To gain a more complete understanding of the information able to be extracted from the number of publications in the top 1% of highly cited papers, three additional parameters are shown in Table 5: the number of publications in the top 0.1% of highly cited papers, the 1% index, and 0.1%∶1% ratio. The 1% index provides the fraction of the total number of publications that reach the top 1% of highly cited papers, which characterizes the size of the tail with reference to the total number of papers. The 0.1%∶1% ratio provides the proportion of the number of publications that reach the top 0.1% of highly cited papers to the number of papers in the top 1% of highly cited papers, which informs the shape of the 1% high-citation tail. Table 5 records these parameters independently for national and multinational papers.

**Table 5:** Characterization of the 1% high-citation tail: the 1% index characterizes the size of the tail and the 0.1%∶1% ratio characterizes the shape of the tail.

	National publications^a^					Multinational publications^a^				
University	Total	1% HC^b^	0.1% HC^b^	1% index^c^	0.1%∶1% ratio^d^	Total	1% HC^b^	0.1% HC^b^	1% index^c^	0.1%∶1% ratio^d^
MIT	2,687	131	22.0	4.9	1.7	1,196	77	11.0	6.4	1.4
Cornell	3,125	70	6.0	2.2	0.9	967	50	5.7	5.2	1.1
Oxford	2,876	50	4.7	1.7	0.9	2,010	89	11.7	4.4	1.3
Kyoto	4,771	40	4.0	0.8	1.0	1,195	45	2.0	3.8	0.4
Toronto	3,943	39	2.0	1.0	0.5	2,309	92	14.3	4.0	1.6
Osaka	5,516	30	3.7	0.5	1,2	1,287	43	7.7	3.3	0.8
Utah	2,085	28	2.0	1.3	0.7	527	18	2.3	3.4	1.3
Stockholm	1,364	14	1.0	1.0	0.7	905	37	8.0	4.1	2.2
Heidelberg	1,649	16	2.0	1.0	1.3	962	37	9.0	3.8	2.4
Seoul National	3,100	22	2.0	0.7	0.9	985	21	3.7	2.1	1.8
Utrecht	1,875	18	1.0	1.0	0.6	1,117	33	2.3	3.0	0.7
ETH Zurich	1,089	14	2.0	1.3	1.4	1,187	28	3.0	2.4	1.1
Melbourne	2,497	19	0.7	0.8	0.4	1,174	33	4.7	2.8	1.4
Sapienza Roma	1,881	9	1.0	0.5	1.1	984	20	2.0	2.0	1.0
Indian Inst. Technology	2,860	5	0.0	0.2	−	614	5	0.0	0.8	−
Paris-Sud 11	1,021	6	1.2	0.6	2.0	823	21	3.0	2.6	1.4
Complutense^e^	1,201	5	0.0	0.4	−	340	5.3	0.3	1.6	0.6
Natl. Auton. México^f^	1,395	1	0.0	0.1	−	789	8	1.7	1.0	2.1

a Means of 2004, 2005, and 2006;

b 1% and 0.1% HC, number of papers in the world’s top 1% and 0.1% of highly cited papers;

c 1% index = 100 times 1% HC divided by the total number of papers;

d 0.1%∶1% ratio = 10 times 0.1% HC divided by 1% HC;

e Complutense University of Madrid;

f National Autonomous University of México.

Although Table 5 represents only a preliminary study to be completed disaggregating the data by research fields (as in Table 1 for chemistry) and with statistical analyses, some clear conclusions can be drawn from these preliminary data. In national publications, the 1% index varied widely across universities, from 4.9 for MIT and 2.2 for Cornell University to 0.2 for the Indian Institute of Technology and 0.1 for the National Autonomous University of México. In comparison to the 1% index, the 0.1%∶1% ratio varied far less, from 2.0 to 0.4, with no apparent association to the values of the 1% index. The values of both parameters did not separate from the nominal mean value of 1.0 very much and varied almost symmetrically above and below this mean value. Multinational publications were notably different, especially in the 1% index, which in 15 out of 18 universities was 2–6 times above the nominal mean value of 1. With the exception of MIT, in which the difference is small, the research performance of universities with the 1% index is assessed far more favorably when considering multinational rather than national publications.

### The h-index does not Distinguish the Highest Citation Percentiles

Unlike other conventional scientometric indicators, the *h*-index is calculated using only the most-cited papers [32]. However, the *h-*index does not correlate with the number of Nobel Prize achievements or with the *x*-index [9] and, as with many other conventional indices, it implies small performance differences between countries and institutions that are research leaders and those at a lower research level [33]. To further investigate why the *h*-index does not provide the expected differences between countries and institutions in research performance, I examined the *h*-core papers from the top- and bottom-ranked universities included in the present study. I first eliminated multinational and review papers, but this modification did not appreciably increase the differences between the *h*-index values for the top and bottom universities. I then examined the percentile positions of the *h*-core papers with respect to citation distribution, for both a single year, 2005, and for the entire 10-year period covered by the ESI database, 2001–2010. The results were clear, while most *h*-core papers were in the top 1% of highly cited papers in top-ranked universities, very few were in the top 1% for bottom-ranked universities.

Returning to the comparison of MIT and Complutense University, their *h*-index values were 115 and 42, respectively, for 2005, when considering only national articles. This indicates an unconvincing research performance ratio of 3∶1. The relevant fact that explains why the *h*-index obscures the differences between these two universities is that while all the MIT *h*-core papers were in the top 1% of highly cited papers, only three out of the 42 *h*-core papers of Complutense University were in the top 1%.

In sum, according to the citation distribution, the *h*-index is much more rigorous for prestigious institutions than for institutions with a lower level of research.

## Discussion

The first question addressed in this study is about the proportion of all scientific publications that are involved in driving the scientific progress. The *x*-index is calculated considering only the papers that are included in the world’s top 1% of cited papers and this amount of papers is very restrictive in comparison to indicators that consider all papers. Surprisingly, a reasonably answer to the posed question is that very few of those in the world’s top 1% of cited papers are really relevant for the progress of science. According with this conclusion the *x*-index predicts the size of a population of papers that is much smaller than the sample of papers from which it is calculated. Especially, the *x-*index was formulated to predict the number of Nobel Prize achievements, which are obviously far less than the 1% of the papers. The best explanation for this prediction is that it is possible because the share of highly cited papers follows a power law [9], in which the frequency of the less frequent events can be predicted from the known frequencies of others that are more frequent, top 1% and 0.1% of highly cited papers.

Additionally, the finding that more than 99% of the published papers do not most likely report on important breakthroughs but do receive more than 90% of the total number of citations strongly suggest that research performance should be evaluated without considering that lower-cited 99% of published papers. If all papers are considered, the real scientific differences between countries and institutions are blurred.

The second part of this study aimed to know the number of papers from universities that are in the world’s top percentiles of highly cited papers and the proportion of multinational, reviews, and MCTS papers. A final aim was to investigate the possibility of simplifying the formula of the *x*-index for universities. For this purpose, the size differences between the 18 universities included in the study are not large and the reported counts may be treated as size-independent.

As a general trend, the rankings of the 18 universities are sound, independently of whether they are based on the counts of papers or citations, either considering the total number of papers or the top 1% of highly cited papers. However, to judge these rankings and to analyze if the ranking parameters accurately reflect the universities’ participation in the progress of science, the variation between the highest and the lowest values of the involved ranking parameter is the most important issue. This variation ranged from 150∶1 for the citation counts of national papers in the top 1% of highly cited papers to 4∶1 for counts of the total number of papers (Tables 2 and 3).

To select a ranking parameter it is worth clarifying that counting papers is better than counting citations if the goal is to rank universities on the basis of their research performance. The reason is statistical: the numbers of papers in the top 1% or 0.1% of highly cited paper for different years are entirely equivalent, which allows to use these counts for statistical analyses. This possibility does not apply to citation counting, unless the number of citations is normalized to correct for the time dependence of citations counting.

Review, MCTS, and multinational papers are all concentrated in the top 1% of highly cited papers. Review papers may be of several types and may be important for fixing knowledge, but they rarely report on important breakthroughs. However, review papers are not as significant a problem because their effect on the indicators for universities seems to be low and they can easily be omitted from counts by restricting database searches. In contrast, MCTS papers cannot currently be identified using bibliometric procedures and therefore cannot be eliminated. For the evaluation of universities the existence of these papers may be ignored, as described above.

Highly cited multinational papers make up a large proportion of the number of papers in the high-citation percentiles of the citation distribution for lower-ranked universities (Table 3). Consequently, in research indicators based on the papers in the high-citation percentiles, multinational papers increase the apparent competitiveness of the lower-ranked universities far more than they do in the case of the higher-ranked universities. Therefore, in these indicators, any errors made in determining what procedure should be applied to rate highly cited multinational papers may mislead the evaluations of lower-ranked universities. In the absence of a convenient counting method for multinational papers their omission from indices based on highly cited papers is an obvious solution [9].

This omission may be controversial because the described effects can potentially be corrected for with fractional counting. In fractional counting, if n countries or institutions participate in producing a paper, each country or institution receives 1/n of the credit [34]–[36]. This procedure is formally correct but does not take into account the fact that, in collaborations between universities from countries with different levels of scientific achievement, the collaborations might not have been symmetrical in terms of scientific leadership and contributions made. This absence of symmetry is suggested both by the proportion of multinational papers in the top 1% of highly cited papers (Table 3), which is larger for the lower-ranked than it is for the higher-ranked universities, and the likelihood that the quality of the research performed by an institution is similar whether it is participating in single-country or multinational papers. This assumption may not be valid, but unless it is demonstrated to be false and scientific contributions made to multinational papers are proven to be strictly equal for all participants, fractional counting cannot be considered the solution to rate multinational collaborations. Fractional counting also may discourage collaborations between highly competitive and less competitive institutions, as the former may wish to avoid lowering their evaluation indices.

Even ignoring these considerations, the crucial point is that the omission of multinational or multi-institutional papers from counts is not a problem when the number of papers remaining in the sample is sufficiently large. In that case, the parameter that best characterizes a country or institution is a size-independent index such as the *z*-index [9]. Once this index is calculated, the total contribution of that country or institution to the advancement of science can be estimated by multiplying the size-independent index by the total number of papers, including the fractional counting of multinational papers. This approach assumes that the scientific leadership of a country or institution is the same in multinational and national papers, as discussed above.

In the present study, I have addressed the problem of multinational papers, but not that of multi-institutional papers. Differences in the scientific performance of countries are perhaps more important for most evaluations than are differences in the scientific performance of different institutions in the same country. However, the elimination of multi-institutional papers in rankings institutions in the same country deserves further investigation.

As a result of these considerations, the formula of the *x* index for universities is:
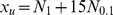
where *N*_1_ and *N*_0.1_ are the number of national articles in the top 1% and 0.1% of highly cited papers, respectively. The *z*_u_ index has the same formula as the *z* index [9] substituting the value of the *x*_u_ index for that of the *x* index. The new formula of the *z*_u_ index eliminating the subtraction term will produce university rankings in which the values of the indicator are 6 units higher than those corresponding to the *z* index. This increase is of little relevance for the high *z* values of the higher-ranked universities but is important for the low *z* values of lower-ranked universities. However, this constant increase does affect rankings and its effect on the indicator is a minor inconvenience considering the current impossibility of bibliometrically eliminating MCTS papers.

The formulas of the *x*_u_ and *z*_u_ indices include the number of national papers in the top 1% and 0.1% most cited papers, but national papers in the top 0.1% of most cited papers are not found among lower-ranked universities (Table 5). This fact precludes that the *x*_u_ and *z*_u_ indices for these universities have exactly the same mathematical meaning as in higher-ranked universities. However, these indices remain sound indicators for ranking universities of low research performance. In the absence of national articles in the top 1% of highly cited papers, in any of the five successive years that are used to calculate the *x* index [9], universities cannot be ranked with the *x*_u_ and *z*_u_ indices. The essential issue that this absence reflects is that these universities have a very low level of research achievement and that they have to be evaluated by their “normal” research activity, e.g. counting all papers or those in the top 10% most cited papers.

I demonstrated, above, that the proportion of *h-*core papers in the 1% high-citation tail is not proportional to the value of the *h*-index. Therefore, using the *h*-index to rank institutions or countries does not provide a clear picture of the differences in research performance among the to-be-ranked institutions or countries. This consideration is much more important for institutions than for researchers. I have asserted in this study that researchers cannot be evaluated using the same indices as are applied to institutions or countries. Researchers are best evaluated by measuring their capability while evaluations of institutions should measure the achievement of important breakthroughs. Because important breakthroughs are low frequency events, the difference is statistical: the achievements of one versus the achievements of 1,000 or more.

Research indicators derived from highly cited papers are field-dependent; the research fields with the highest citation rates have the highest influence in the indicators of multidisciplinary institutions such as universities. This problem is common with any index for institutions and journals, and can be solved by analyzing each paper in its corresponding field of research (as has been done in Figure 1 and Table 1). It is also possible to normalize the number of citations across fields (see, for example, [34], [37]–[40]). However, applying this approach introduces potential risks in that institutional rankings then depend on the number of references in each paper, which in turn depends on authors’ behavior, which in turn might be influenced by the evaluation process. Experience with the impact factor (e.g., [41]) shows that scientific evaluations must estimate research performance in a way that depends as little as possible on authorial decisions that are not related to research goals because unintended consequences are likely to occur [6], [7].
